# Vibe coding with preservice teachers: a single-group pretest-posttest pilot study of programming self-efficacy and intentions to use generative AI in education

**DOI:** 10.3389/fpsyg.2026.1957111

**Published:** 2026-09-28

**Authors:** Kağan Kırcaburun

**Affiliations:** Educational Sciences Department, Duzce Universitesi Egitim Fakultesi, Düzce, Türkiye

**Keywords:** artificial intelligence, educational generative AI, preservice teachers, programming self-efficacy, vibe coding

## Abstract

**Introduction:**

Generative artificial intelligence (GAI) is changing how novices engage with programming by enabling program creation through natural-language interaction, an approach increasingly described as vibe coding. This may be particularly relevant for preservice teachers, who are often expected to develop computational skills despite limited programming experience. Grounded in social cognitive theory, this study examined changes in programming self-efficacy and intentions to use GAI in education following a four-week vibe coding intervention.

**Methods:**

A single-group pretest-posttest design was used with 159 preservice teachers (92.5% women) from four undergraduate programs at a Turkish state university. The intervention combined instructor demonstrations with individual programming tasks completed through natural-language prompting. Programming self-efficacy for simple and complex tasks and, as a secondary outcome, intentions to use GAI in education were assessed before and after the intervention.

**Results:**

Because full measurement invariance was not established, pretest-posttest differences were interpreted as changes in observed scores. Significant increases were found for simple-task self-efficacy (Cohen’s dz = 2.62), complex-task self-efficacy (Cohen’s dz = 1.72), and intentions to use GAI in education (Cohen’s dz = 0.29), with all comparisons remaining significant after Bonferroni correction.

**Discussion:**

The findings provide preliminary evidence that structured vibe coding activities may be associated with greater programming-related confidence among preservice teachers. However, the absence of a control group prevents attribution of the observed changes to the intervention, and the large self-efficacy effects may partly reflect very low pretest scores and regression to the mean. In addition, the CPSES measures general programming confidence rather than unaided programming ability, so posttest gains may partly reflect confidence in completing tasks with GAI support. Future studies should use controlled designs and performance-based measures to determine whether increased confidence is accompanied by gains in independent programming competence.

## Introduction

Generative artificial intelligence (GAI) has become increasingly prominent in education, with large language models capable of generating text, feedback, and code in response to natural-language prompts (Kasneci et al., 2023). Research on GAI in education has expanded accordingly, although much of this work has focused on student use and institutional adoption rather than on preparing the educators who will ultimately shape how these tools are used in classrooms (Crompton and Burke, 2023; Zawacki-Richter et al., 2019). Teachers occupy a central role in this process because the educational value of technology depends substantially on their knowledge, beliefs, and willingness to integrate it into instruction (Celik, 2023; Ertmer and Ottenbreit-Leftwich, 2010). Preservice teachers are particularly important in this regard because their current beliefs and competencies may influence how future students encounter GAI-supported learning (Celik, 2023; Ertmer and Ottenbreit-Leftwich, 2010).

At the same time, expectations regarding teachers’ computational competencies have increased (Yadav et al., 2014). Computational thinking is widely regarded as an important educational competency that teachers across subject areas may be expected to model and support (Grover and Pea, 2013; Yadav et al., 2014), and teacher education programs have increasingly been encouraged to introduce programming-related experiences to preservice teachers regardless of disciplinary background (Yadav et al., 2014). However, most preservice teachers outside computing-related fields have little or no programming experience, and traditional syntax-based instruction can present a substantial barrier (Lye and Koh, 2014; Yadav et al., 2014). Early difficulties with programming syntax, debugging, and error interpretation may undermine confidence before meaningful learning experiences emerge (Lye and Koh, 2014; Prather et al., 2023). For these learners, affective experiences may be as important as cognitive ones during the initial stages of programming education (Ertmer and Ottenbreit-Leftwich, 2010; Scherer et al., 2019).

Programming self-efficacy—an individual’s belief in their capability to accomplish programming tasks—is central to this affective dimension (Compeau and Higgins, 1995). Self-efficacy theory suggests that beliefs about one’s capabilities influence engagement, persistence, and performance (Bandura, 1977, 1997). In educational settings, self-efficacy is among the most consistent correlates of academic engagement and achievement (Pajares, 1996; Zimmerman, 2000), and domain-specific measures of computer and programming self-efficacy have long been associated with persistence and performance in computing courses (Compeau and Higgins, 1995). Importantly, self-efficacy develops primarily through mastery experiences and vicarious experiences, alongside social persuasion and physiological states (Bandura, 1997; Usher and Pajares, 2008). Consequently, interventions that provide opportunities for early success may be particularly beneficial for learners with limited confidence in their programming abilities (Compeau and Higgins, 1995; Usher and Pajares, 2008).

Recent advances in GAI have altered what programming can look like for novices (Prather et al., 2023). Code-generating models can translate natural-language descriptions into executable programs and have demonstrated strong performance on introductory programming tasks (Finnie-Ansley et al., 2022). Building on this capability, vibe coding has emerged as an approach to GAI-assisted programming in which users describe the intended behavior of a program in everyday language, the GAI system generates candidate code, and users iteratively refine the output through further prompts (Elgendy et al., 2026; Munoz and Rook, 2026). Rather than focusing primarily on syntax construction, this workflow places greater emphasis on describing goals, evaluating outputs, and refining prompts (Elgendy et al., 2026; Kshetri and Voas, 2026). Programming activity is therefore organized around interaction with GAI systems rather than line-by-line code production (Meske et al., 2025; Munoz and Rook, 2026).

This interaction structure differs from other forms of GAI-assisted programming. In typical GAI pair-programming and code-completion settings, users remain programmers in the conventional sense because GAI-generated suggestions must still be reviewed, understood, and integrated into the program (Prather et al., 2023). In vibe coding, by contrast, natural language becomes the primary medium of interaction, and greater emphasis is placed on problem formulation and evaluation of outputs rather than on syntactic fluency (Meske et al., 2025; O'Connor et al., 2026). Debugging likewise occurs largely at the level of intended behavior, with users revising descriptions and prompts rather than locating syntax errors (O'Connor et al., 2026). For complete novices, this reorganization may be particularly important because it removes a barrier that often discourages learners during the early stages of programming instruction (Munoz and Rook, 2026; O'Connor et al., 2026).

These approaches can be distinguished by how much responsibility remains with the learner and by the main form of interaction. In code completion, the learner writes the program while the model fills in fragments, so syntactic knowledge and source-level debugging remain central (Finnie-Ansley et al., 2022; Prather et al., 2023). GAI pair programming also preserves substantial learner control, with the model supporting explanation and code revision while the human remains responsible for understanding and integrating the output (Prather et al., 2023). Prompt-based programming moves further toward natural-language interaction, but users are still expected to verify the implementation at the code level. Vibe coding differs by delegating more of the implementation to the model, with users expressing intent and judging the result mainly by how the program behaves (Elgendy et al., 2026; Kshetri and Voas, 2026; Meske et al., 2025). In educational settings, however, vibe coding is usually scaffolded rather than fully autonomous (Pellas, 2026). The present intervention follows this scaffolded form, and the term vibe coding is used throughout in that instructional sense.

The elements that remain central in vibe coding are closely aligned with core components of computational thinking emphasized in teacher education (Grover and Pea, 2013; Yadav et al., 2014). At the same time, the approach is not without risks. When learners accept generated code without sufficient evaluation, GAI assistance may encourage overreliance or a perceived sense of competence that is not always accompanied by deeper understanding (Kırcaburun, 2026; Prather et al., 2023). Whether vibe coding functions as an accessible entry point or a learning shortcut may therefore depend on instructional design (Munoz and Rook, 2026). In particular, learners may benefit most when instructional activities encourage active engagement with the problem-solving process rather than passive acceptance of generated solutions (Munoz and Rook, 2026; Prather et al., 2023). The present intervention was designed with this consideration in mind, combining instructor-modeled prompting and debugging with cumulative individual assignments that required students to produce functioning programs.

From the perspective of social cognitive theory, vibe coding may be a promising approach for supporting programming self-efficacy among novice learners. Because learners can produce working programs from the outset, the approach may provide opportunities for mastery experiences, while instructor demonstrations of prompting and debugging may provide complementary vicarious experiences (Bandura, 1997; Usher and Pajares, 2008). A small number of recent intervention studies have begun to examine this question, but the findings remain mixed. Emirtekin et al. (2026) reported increased programming self-efficacy after a four-week vibe coding intervention with 50 undergraduate students, whereas Pellas (2026) observed a decline in self-reported coding and teaching confidence among 24 preservice teachers and interpreted this as a recalibration of expectations. Kang and Huang (2026) also found higher coding-related self-efficacy after a GAI-supported module, although these gains were not significantly related to coding performance. Together, these findings suggest that GAI-supported programming may influence confidence in different ways depending on the learning context and how self-efficacy is interpreted.

This issue may be especially relevant for preservice teachers outside computing-related programs, whose programming self-efficacy tends to be lower despite increasing expectations to engage with GAI-supported educational technologies (Aksoğan et al., 2020; Celik, 2023; Yadav et al., 2014). The present study addresses this gap with a larger sample than previous intervention studies and a workflow in which programming tasks were completed entirely through natural-language prompting.

A second relevant outcome is preservice teachers’ intention to use GAI in their future educational practice. Behavioral intention is among the most proximal predictors of technology use in established models of technology acceptance (Davis, 1989; Venkatesh et al., 2003), and evidence from teacher education suggests that self-efficacy-related beliefs are associated with technology acceptance and intended use (Scherer et al., 2019). Recent research similarly indicates that teachers’ professional knowledge and beliefs are associated with intentions to integrate GAI into education (Celik, 2023), whereas studies in higher education suggest that confidence-related factors are associated with acceptance of GAI tools (Chan and Hu, 2023; Strzelecki, 2024). Accordingly, direct experience using GAI to create functioning programs may be associated with stronger intentions to use such tools in future teaching, although high baseline intentions may limit the extent of observable change.

Accordingly, the present study examined a four-week vibe coding intervention with preservice teachers in which weekly instructor-led demonstrations provided vicarious experiences and cumulative individual assignments provided opportunities for mastery experiences. Two hypotheses were tested. First, programming self-efficacy for both simple and complex programming tasks was expected to increase from pretest to posttest (H1). Second, intentions to use GAI in education were expected to increase from pretest to posttest (H2).

## Methods

### Participants and procedure

Participants were recruited from intact classes enrolled in an elective undergraduate course. A single-group pretest-posttest design was adopted because random assignment was not feasible within the existing course structure. Participation was voluntary, and verbal informed consent was obtained before data collection. Participants received standardized information about the study and were informed that they could decline or withdraw without any effect on their course standing. Written consent was not collected because the procedure approved by the institutional ethics committee specified verbal informed consent. Because the design required pretest and posttest responses to be matched at the individual level, participants wrote their names on the pretest questionnaire, and the same questionnaires were redistributed to them by name at the posttest administration, so that the two observations could be matched directly. Identifying information was used solely for this matching procedure and was not included in the dataset used for statistical analysis or in any reported results. Completion of the programming assignments formed part of the course assessment. Grading was based solely on assignment submission, as evidenced by screenshots documenting functioning programs, and was unrelated to assignment quality or responses to the study measures. To minimize the influence of the assessment context on responding, participants were informed that questionnaire responses would be used only for research purposes and would not affect their course grades.

Because the study was conducted by a single author, the same person served as both instructor and researcher. This dual role may have introduced demand characteristics or expectancy effects, particularly because participants were enrolled in the instructor’s course. Several procedural safeguards were used to reduce this risk. Although identifying information was recorded so that the pretest and posttest questionnaires could be matched, individual questionnaire responses were not reviewed by the instructor during the intervention, and grades were based only on assignment submission rather than the quality of the work. Participants were also informed that their responses would not affect their course standing. However, no independent observer or manipulation check was used, so expectancy effects cannot be ruled out as a possible contributor to the observed changes. A total of 187 students were enrolled in the course. However, 28 students (15.0% of those enrolled) were excluded because they did not provide both pretest and posttest data. In most cases, this resulted from absence during one of the two administrations rather than withdrawal from the course. Only complete paired observations were entered into the dataset, and no records were retained for students who did not provide both administrations. No individual-level information is therefore available for the excluded students, and it was not possible to compare them with the analyzed sample or to determine whether the missing paired observations were systematically related to participant characteristics.

The final sample comprised 159 preservice teachers enrolled in a faculty of education at a state university in Türkiye. Participants included 147 women (92.5%) and 12 men (7.5%) from four undergraduate programs: primary education (*n* = 49, 30.8%), early childhood education (*n* = 43, 27.0%), psychological counseling and guidance (*n* = 35, 22.0%), and Turkish language education (*n* = 32, 20.1%). Most participants reported using GAI primarily for educational purposes (54.1%) or general information seeking (40.9%). Daily GAI use was generally low, with most participants reporting less than 1 h (55.4%) or between one and 2 h (34.0%) of use per day (see Table 1). The study protocol was approved by the relevant institutional ethics committee (Document No: E-78187535-050.04-712917) and was conducted in accordance with the Declaration of Helsinki. The pretest was administered during the first week of the intervention, and the posttest was administered during the final session using the same self-report measures.

**Table 1:** Participants’ demographic characteristics.

Variable	*N*	%
Gender		
Women	147	92.5
Men	12	7.5
Department		
Primary education	49	30.8
Early childhood education	43	27.0
Psychological counseling	35	22.0
Turkish language education	32	20.1
GAI use purpose		
Education	86	54.1
General knowledge	65	40.9
Social interaction	8	5.0
Daily GAI use duration		
Less than 1 h	88	55.4
Between 1 and 2 h	54	34.0
Between 2 and 3 h	10	6.3
Between 3 and 4 h	5	3.1
More than 4 h	2	1.3

GAI, generative artificial intelligence.

### The vibe coding intervention

The intervention was designed to introduce preservice teachers to vibe coding, an approach in which users create programs through natural-language interaction with a generative AI system rather than by writing code manually (Elgendy et al., 2026; Munoz and Rook, 2026). The approach was considered appropriate for participants with limited programming experience because it enabled them to develop and test programs without requiring prior knowledge of programming syntax. The intervention was informed by social cognitive theory and was structured around weekly instructor demonstrations, guided in-class practice, and independent practice activities (see Figure 1).

**Figure 1:**
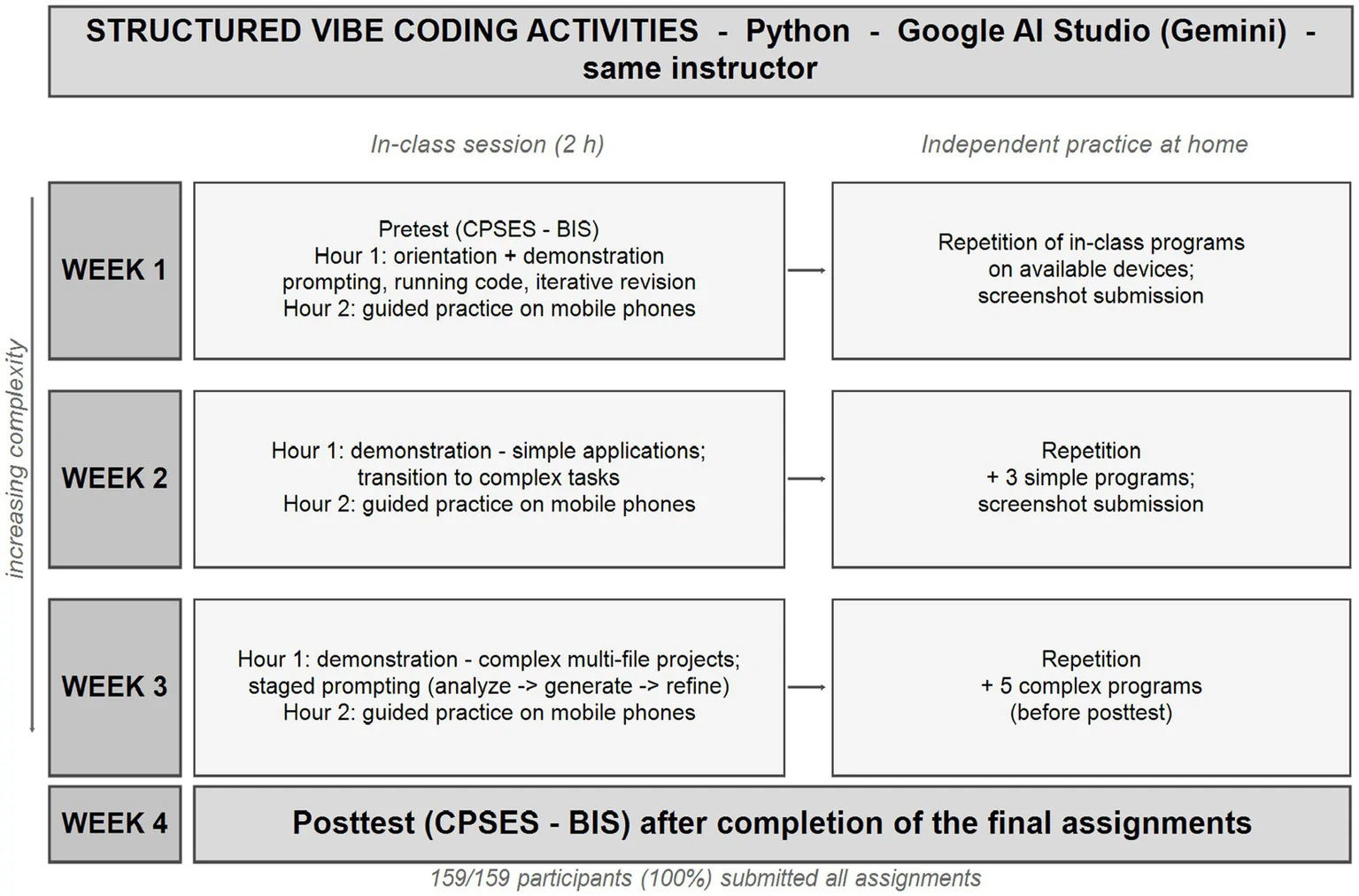
Overview of the four-week vibe coding intervention. Weeks 1–3 each combined instructor-led demonstration (vicarious experience) with guided in-class practice on mobile phones and independent home practice (mastery experiences); program complexity increased across weeks. CPSES, Computer Programming Self-Efficacy Scale; BIS, Behavioral Intention Scale.

The intervention was delivered across four consecutive weeks within a regularly scheduled elective undergraduate course titled Artificial Intelligence Applications in Education. All sessions were conducted by the same instructor. The pretest was administered at the beginning of the first session, and the instructional component began later in the same session and continued across two subsequent weekly two-hour sessions, yielding approximately six instructional hours in total. The posttest was administered during the fourth week after completion of the final assignments. Programming activities were completed in Python using Google AI Studio through a standard web browser. Participants used the Gemini 2.5 Flash model with default settings and no custom system instructions, allowing them to reproduce the same environment independently outside class.

Each instructional session followed the same structure. During the first hour, the instructor demonstrated how to formulate prompts, evaluate generated outputs, and revise prompts when the program did not function as intended. During the second hour, participants reproduced and extended the demonstrated programs on their own mobile devices while receiving guidance from the instructor. Because the classroom did not contain computers, access to the browser-based GAI environment via mobile devices enabled supervised hands-on programming activities without requiring a computer laboratory.

Instructional content increased in complexity across the three teaching weeks. The first session introduced vibe coding and prompt formulation through simple programming tasks, such as basic calculations and formatted output. The second session consolidated these skills and introduced more demanding applications. The third session focused on larger and more complex projects and introduced a staged prompting strategy in which participants first used GAI to analyze a problem and specify intended functionality before generating and refining code. Activities progressed from short programs to multi-component applications requiring data entry, storage, analysis, and recommendation functions.

After each instructional session, participants repeated the in-class activities independently and completed additional assignments. Following the second session, participants completed three simple programming tasks, whereas five more complex tasks were assigned after the third session. For each assignment, participants executed the program, documented the functioning output with screenshots, and submitted the material electronically. All participants submitted the required assignments. These submissions were used to verify engagement with the practical component of the intervention rather than to evaluate programming performance. The combination of instructor-guided activities and repeated independent practice was intended to provide participants with multiple opportunities to develop confidence in their ability to create functioning programs.

To support replication, the full instructional materials are provided as Supplementary material. Supplementary material S1 presents the instructor prompts used across the sessions, including the staged prompting strategy introduced in Week 3. Supplementary material S2 provides the eight assignments and the criteria used to record completion, while Supplementary material S3 includes representative student submissions. Assignment completion was coded on a binary basis when the submitted screenshot showed a functioning program that produced the required output. No separate rubric for code quality was used. Because submissions consisted of screenshots rather than interaction logs, prompt frequency and the extent of students’ revisions to GAI-generated code could not be examined. These limitations are addressed further in the limitations section.

### Measures

Computer Programming Self-Efficacy Scale (CPSES): Programming self-efficacy was assessed using the CPSES (Altun and Mazman, 2012). The scale comprises nine items assessing simple programming tasks (3 items; e.g., “I can write a program that calculates the average of three numbers.”) and complex programming tasks (6 items; e.g., “I can organize and design a program in a modular manner.”). Participants rated each item on a 7-point Likert scale ranging from 1 (not confident at all) to 7 (completely confident), with higher scores indicating greater programming self-efficacy. In the present study, internal consistency was high for both simple programming self-efficacy (α = 0.92/0.95, ω = 0.92/0.95) and complex programming self-efficacy (α = 0.85/0.91, ω = 0.88/0.92) at pretest and posttest.

Behavioral Intention Scale (BIS): Intentions to use GAI in education were assessed using the BIS (Yurt and Kaşarcı, 2025). The scale comprises five items assessing preservice teachers’ intentions to use GAI technologies in their future educational practice. An example item is, “I will pay more attention to emerging GAI applications for my professional development.” Responses are provided on a 5-point Likert scale ranging from 1 (completely false) to 5 (completely true), with higher scores indicating stronger intentions to use GAI in education. In the present study, internal consistency was high at both pretest and posttest (α = 0.89/0.86, ω = 0.89/0.87).

### Data analytic strategy

All analyses were conducted using IBM SPSS Statistics and AMOS. Descriptive statistics were calculated for all study variables, including means, standard deviations, skewness, and kurtosis values. Following commonly used guidelines, skewness and kurtosis values within ±1 were considered indicative of acceptable univariate normality (Kline, 2016). In addition, change-score distributions (posttest-pretest) were visually inspected using histograms to evaluate the normality of pretest-posttest differences. Internal consistency was evaluated using Cronbach’s alpha (α) and McDonald’s omega (ω). Convergent validity was assessed using average variance extracted (AVE) and composite reliability (CR). AVE values of 0.50 or higher and CR values of 0.70 or higher were considered indicative of acceptable convergent validity and construct reliability (Fornell and Larcker, 1981).

The factorial validity of the Computer Programming Self-Efficacy Scale (CPSES) and the Behavioral Intention Scale (BIS) was examined using confirmatory factor analysis (CFA). Model fit was evaluated using multiple fit indices, including the chi-square to degrees-of-freedom ratio (χ^2^/df), the comparative fit index (CFI), the goodness of fit index (GFI), the standardized root mean square residual (SRMR), and the root mean square error of approximation (RMSEA). Model fit was evaluated using commonly recommended criteria. Values below 5 for χ^2^/df, 0.90 or higher for CFI and GFI, and below 0.08 for SRMR and RMSEA were considered acceptable (Byrne, 2010; Hu and Bentler, 1999). The chi-square statistic and degrees of freedom are reported together with the fit indices, while standardized factor loadings are provided in Supplementary material S4 to support interpretation of the measurement models.

Because the same instruments were administered at pretest and posttest, longitudinal measurement invariance was examined before interpreting score changes. Corresponding item residuals were allowed to correlate across occasions, and configural, metric, and scalar models were tested sequentially (Little, 2013). Changes of no more than 0.010 in CFI and 0.015 in RMSEA were used as criteria for invariance (Cheung and Rensvold, 2002). Partial invariance was not pursued because freeing parameters based on modification indices within the same sample would be exploratory and would require independent cross-validation. Full model results are provided in Supplementary material S5.

Pretest-posttest changes in programming self-efficacy and intentions to use GAI in education were examined using paired-samples t-tests. Cohen’s dz. was calculated to estimate effect sizes, with values of 0.20, 0.50, and 0.80 representing small, medium, and large effects, respectively (Cohen, 1988). To evaluate the robustness of the findings, Wilcoxon signed-rank tests were also conducted. Effect sizes for the Wilcoxon analyses were calculated, with values of 0.10, 0.30, and 0.50 representing small, medium, and large effects, respectively (Field, 2018).

Three pretest-posttest comparisons were conducted, corresponding to the two hypotheses specified in advance of data collection. Because the outcomes were pre-specified rather than selected after inspection of the data, a correction for multiplicity was not strictly required; nevertheless, Bonferroni-corrected values (α = 0.05/3 = 0.017) and Benjamini-Hochberg adjusted values at q = 0.05 are reported in Supplementary material S6 (Benjamini and Hochberg, 1995).

## Results

### Construct validity

Confirmatory factor analyses were conducted separately for the Computer Programming Self-Efficacy Scale (CPSES) and the Behavioral Intention Scale (BIS) at pretest and posttest (Table 2). Overall, fit indices varied across instruments and measurement occasions. For the CPSES, the pretest model yielded χ^2^(23) = 97.97, χ^2^/df = 4.26, SRMR = 0.06, and CFI = 0.92, while the posttest model showed χ^2^(23) = 50.94, χ^2^/df = 2.22, SRMR = 0.06, and CFI = 0.98. For the BIS, the values were χ^2^(4) = 5.96, χ^2^/df = 1.49, SRMR = 0.02, and CFI = 0.99 at pretest, and χ^2^(4) = 14.25, χ^2^/df = 3.56, SRMR = 0.04, and CFI = 0.97 at posttest. Standardized factor loadings for both occasions are reported in Supplementary Table S4.2. RMSEA values were elevated in three of the four models, and the CPSES pretest model also fell below the GFI criterion. Researchers have noted that RMSEA may overestimate model misfit when models contain relatively few degrees of freedom, particularly in studies with modest sample sizes. Under these circumstances, greater emphasis is often placed on alternative fit indices such as CFI and SRMR when evaluating model adequacy (Kenny et al., 2015). Taken together, the CFA findings provide only partial support for the expected factor structures. Of the four models, only the BIS pretest model met all predefined fit criteria. The CPSES pretest model exceeded the RMSEA threshold and fell slightly below the GFI criterion, while the CPSES and BIS posttest models also showed elevated RMSEA values. Although the low degrees of freedom may partly explain these results, this interpretation should be considered alongside the item-level evidence. All standardized factor loadings exceeded 0.60 at both occasions, with the lowest loading observed for the item concerning maintaining focus despite distractions. Overall, the measurement models showed acceptable but not consistently strong fit, and factorial validity should therefore be interpreted cautiously rather than as fully established.

**Table 2:** CFA model fit indices, along with recommended cut-off values.

Fit indices	Reference values	Pretest		Posttest	
		CPSES	BIS	CPSES	BIS
χ^2^ /df	≤ 5 (Byrne, 2010)	4.26	1.49	2.22	3.56
RMSEA	≤ 0.08 (Hu and Bentler, 1999)	0.14	0.06	0.09	0.13
SRMR	≤ 0.08 (Byrne, 2010)	0.06	0.02	0.06	0.04
CFI	≥ 0.90 (Hu and Bentler, 1999)	0.92	0.99	0.98	0.97
GFI	≥ 0.90 (Hu and Bentler, 1999)	0.89	0.99	0.94	0.97

CFA, Confirmatory factor analysis; χ^2^ = chi-square statistic; df = degrees of freedom; CFI, comparative fit indices; GFI, goodness of fit index; RMSEA, root mean square error of approximation; CPSES, Computer Programming Self-Efficacy Scale; BIS, Behavioral Intention Scale.

### Longitudinal measurement invariance

Longitudinal measurement invariance was examined for both instruments, with full results reported in Supplementary Table S5. For the CPSES, the configural model showed inadequate fit (χ^2^(120) = 351.01, CFI = 0.901, TLI = 0.873, RMSEA = 0.110), preventing further invariance testing. For the BIS, the configural model showed acceptable CFI and TLI values, although RMSEA remained elevated (χ^2^(29) = 78.32, CFI = 0.942, TLI = 0.910, RMSEA = 0.104). Metric invariance was supported (ΔCFI = +0.002, ΔRMSEA = −0.009), but scalar invariance was not (ΔCFI = −0.015, ΔRMSEA = +0.005). Full measurement equivalence was therefore not established for either instrument. Accordingly, the pretest-posttest results are reported as changes in observed scores and should be interpreted cautiously because participants may not have interpreted all items in the same way across occasions.

### Descriptive statistics and reliability

Table 3 presents descriptive statistics and measurement properties for the study measures at pretest and posttest. For simple programming self-efficacy, AVE values were 0.79 and 0.86 and composite reliability (CR) values were 0.92 and 0.95 at pretest and posttest, respectively. For complex programming self-efficacy, AVE values were 0.52 and 0.64 and CR values were 0.87 and 0.91. Intentions to use GAI in education also demonstrated satisfactory convergent validity, with AVE values of 0.63 and 0.55 and CR values of 0.89 and 0.85 at pretest and posttest, respectively. Overall, the AVE and CR values provided support for the convergent validity of the study measures.

**Table 3:** Descriptive statistics and measurement properties of the study measures at pretest and posttest.

Items	Pretest	Posttest	Pretest	Posttest
	M (SD)		AVE (CR)	
Programming self-efficacy (CPSES)				
Simple tasks				
I can write a program that displays the message “Hello, World!”	1.50 (1.00)	5.58 (1.49)	0.79 (0.92)	0.86 (0.95)
I can write a program that calculates the average of three numbers.	1.48 (0.90)	5.72 (1.39)		
I can write a program that calculates the average of any given sequence of numbers.	1.46 (0.94)	5.72 (1.46)		
Complex tasks				
I can write a fairly complex and lengthy program to solve a problem when the requirements are clearly defined.	1.23 (0.55)	4.19 (1.59)	0.52 (0.87)	0.64 (0.91)
I can organize and design a program in a modular manner.	1.38 (0.86)	3.88 (1.41)		
I can debug a long and complex program and make it fully functional.	1.32 (0.80)	3.64 (1.46)		
I can understand a long, complex program that consists of multiple files.	1.70 (1.19)	3.65 (1.50)		
I can rewrite long and complex sections of a program to make it more readable and easier to understand.	1.55 (1.01)	3.84 (1.63)		
I can find ways to stay focused on programming even when there are many distractions around me.	2.13 (1.51)	4.33 (1.52)		
Behavioral intention (BIS)				
I will continue to learn about AI knowledge for my professional teaching life.	4.25 (0.81)	4.37 (0.65)	0.63 (0.89)	0.55 (0.85)
I will keep myself updated with the latest AI applications for my Professional development.	4.18 (0.74)	4.36 (0.65)		
I plan to spend time in learning AI technology in the future to enhance my teaching quality.	3.84 (0.93)	4.16 (0.75)		
I will pay more attention to emerging AI applications for my Professional development.	4.08 (0.78)	4.30 (0.69)		
I intend to use AI to assist my teaching.	4.35 (0.82)	4.49 (0.68)		

M, mean; SD, standard deviation; AVE, average variance extracted; CR, composite reliability; CPSES, Computer Programming Self-Efficacy Scale; BIS, Behavioral Intention Scale.

At baseline, participants reported very low levels of programming self-efficacy for both simple (M = 1.48, SD = 0.88) and complex (M = 1.55, SD = 0.78) programming tasks (Table 4). Following the intervention, mean scores increased to 5.67 (SD = 1.37) for simple programming self-efficacy and 3.92 (SD = 1.27) for complex programming self-efficacy. Intentions to use GAI in education were comparatively high at pretest (M = 4.14, SD = 0.68) and increased modestly at posttest (M = 4.34, SD = 0.55).

**Table 4:** Paired-samples t-test results for pretest-posttest comparisons.

Construct	Pretest M (SD)	Posttest M (SD)	Mean difference	t (df)	p	Cohen’s dz
Simple tasks	1.48 (0.88)	5.67 (1.37)	4.19 (1.60)	33.05 (158)	<0.001	2.62
Complex tasks	1.55 (0.78)	3.92 (1.27)	2.37 (1.38)	21.64 (158)	<0.001	1.72
Behavioral intention	4.14 (0.68)	4.34 (0.55)	0.20 (0.68)	3.68 (158)	<0.001	0.29

### Pretest-posttest comparisons

Prior to the main analyses, skewness and kurtosis values were inspected and all values fell within the ±1 range (not depicted as table), providing support for the assumption of normality. In addition, change-score distributions (posttest-pretest) were examined visually using histograms (Figure 2). The distributions for simple programming self-efficacy, complex programming self-efficacy, and intentions to use GAI in education were broadly consistent with normality. Although simple programming self-efficacy exhibited a slight concentration of scores at the upper end of the distribution, substantial departures from normality were not observed. Therefore, paired-samples t-tests were used to examine pretest-posttest changes. As a robustness check, Wilcoxon signed-rank tests were also conducted.

**Figure 2:**
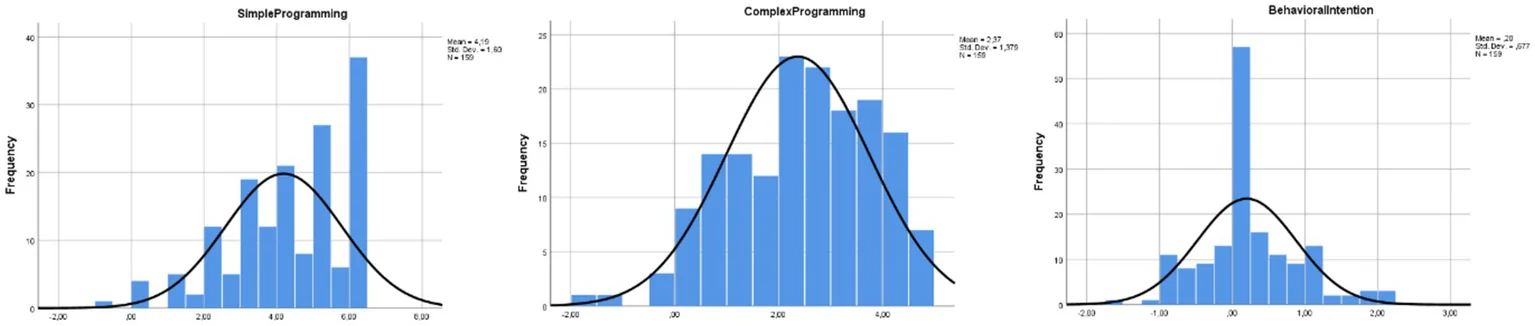
Histograms depicting change scores (posttest-pretest) for simple programming tasks, complex programming tasks, and behavioral intention suggested that the distributions were broadly consistent with normality. While simple programming tasks exhibited a slight concentration of scores at the upper end of the distribution, overall deviations from normality appeared minimal.

Paired-samples *t*-tests indicated significant increases across all outcomes (Table 4). Simple programming self-efficacy increased significantly from pretest to posttest, t(158) = 33.05, *p* < 0.001, 95% CI [3.94, 4.44], corresponding to a very large effect size (Cohen’s dz. = 2.62). Complex programming self-efficacy also increased significantly, t(158) = 21.64, *p* < 0.001, 95% CI [2.15, 2.58], with a very large effect size (Cohen’s dz. = 1.72). Intentions to use GAI in education showed a smaller but statistically significant increase, t(158) = 3.68, *p* < 0.001, 95% CI [0.09, 0.30], corresponding to a small effect size (Cohen’s dz. = 0.29). All three comparisons remained significant under Bonferroni correction and under Benjamini-Hochberg false discovery rate control at q = 0.05 (Supplementary Table S6), so the substantive conclusions do not depend on how multiplicity is handled.

The robustness analyses yielded the same pattern of findings (see Table 5). Wilcoxon signed-rank tests demonstrated significant increases in simple programming self-efficacy (Z = 10.85, *p* < 0.001, r = 0.86), complex programming self-efficacy (Z = 10.60, p < 0.001, r = 0.84), and intentions to use GAI in education (Z = 3.35, *p* = 0.001, r = 0.27). These results indicate that the observed pretest-posttest improvements were not dependent on the assumptions of parametric testing.

**Table 5:** Wilcoxon signed-rank test results for pretest-posttest comparisons.

Variable	N	Wilcoxon W	Z	p	r
Programming self-efficacy (simple tasks)	159	12244.00	10.85	< 0.001	0.86
Programming self-efficacy (complex tasks)	159	11841.50	10.60	< 0.001	0.84
Intention to use GAI in education	159	4981.00	3.35	0.001	0.27

## Discussion

The present study examined whether a four-week vibe coding intervention was associated with changes in programming self-efficacy and intentions to use GAI in education among preservice teachers. Three findings stand out. First, programming self-efficacy scores were substantially higher at posttest than at pretest, with large effects for both simple and complex tasks. Second, the increase was larger for simple tasks than for complex tasks, and posttest confidence for complex tasks remained near the midpoint of the scale. Third, intentions to use GAI in education increased significantly but modestly from an already high pretest baseline. Taken together, these findings provide preliminary evidence that vibe coding may offer an accessible entry point to programming for learners without a computing background, while also highlighting the limits of a brief intervention.

Before interpreting these findings, the correspondence between the intervention and the outcome measure should be considered. Participants completed programming tasks through natural-language interaction with a GAI system, whereas the CPSES assesses general programming capabilities without referring to GAI support. Posttest scores should therefore not be interpreted as evidence of improved unaided programming capability. At least part of the increase may reflect greater confidence in completing programming tasks with GAI assistance, which was the form of programming practiced during the intervention. However, the construct cannot be relabeled as GAI-assisted programming self-efficacy because the CPSES was not designed to measure this distinction and participants were not asked how they interpreted the items. The findings are therefore reported as changes in CPSES scores, with the possibility that item interpretation shifted across occasions. This concern is also consistent with the invariance results, which did not establish full measurement equivalence. Future research should assess unaided and GAI-assisted programming self-efficacy separately using measures developed for each context.

The self-efficacy findings are consistent with the social cognitive framework that informed the intervention design. Self-efficacy theory proposes that mastery experiences are a primary source of efficacy beliefs and that vicarious experiences can further support their development (Bandura, 1997; Usher and Pajares, 2008). The intervention was designed to provide both sources of self-efficacy. Weekly instructor demonstrations offered vicarious experiences through modeled prompting and debugging, while cumulative assignments provided opportunities for participants to produce functioning programs themselves. For participants whose pretest confidence was very low, repeated success in producing functioning programs may have provided the kinds of experiences that self-efficacy theory identifies as important for developing confidence (Bandura, 1977; Pajares, 1996). In this respect, the findings extend previous research linking successful programming experiences with computing-related confidence (Compeau and Higgins, 1995) to the context of GAI-supported programming and vibe coding (O'Connor et al., 2026; Prather et al., 2023).

At the same time, the magnitude of these gains should be interpreted in light of the starting point. Pretest means below 1.6 on a 7-point scale left substantial room for improvement, and some portion of the large effect sizes likely reflects these low baseline levels, as noted in the limitations. A more conservative interpretation is that participants moved from very low to moderate-to-high programming confidence over the four-week period. Regression to the mean is particularly relevant because pretest scores were close to the lower end of the response scale, making upward movement on repeated measurement more likely. The large effect sizes (dz = 2.62 and 1.72) should therefore not be interpreted as estimates of the effect of vibe coding. Emirtekin et al. (2026), using the same instrument in a comparable four-week single-group intervention with higher baseline scores, reported a smaller increase (d = 0.93), which is consistent with baseline differences contributing to the larger effects observed here. Other influences associated with the single-group design, including repeated measurement and concurrent course experiences, also cannot be separated from any contribution of the intervention. From a practical perspective, this shift may be more important than the precise magnitude of the standardized effects, as increased self-efficacy is an essential motive to learn that consistently predicts students’ choice of activities, effort, and persistence (Zimmerman, 2000).

The difference between simple and complex task self-efficacy is informative in its own right. Posttest confidence for simple tasks approached the upper end of the scale, whereas confidence for complex programming tasks remained closer to the midpoint. This pattern is theoretically consistent with self-efficacy theory, which suggests that mastery experiences are most influential for tasks that resemble those previously mastered (Bandura, 1997). The intervention provided repeated opportunities to complete relatively short programs but fewer opportunities to engage with more complex programming tasks. The findings therefore suggest that initial confidence may be easier to develop for simple than for more advanced programming tasks, and that confidence for more advanced tasks may require longer or more extensive experience. For teacher education, this implies that short interventions may serve as an entry point rather than a complete preparation for computational work (Yadav et al., 2014).

This pattern also aligns with the interaction structure of vibe coding. Simple programs can often be described through a single prompt, making successful outcomes relatively easy to achieve once the syntax barrier is removed (Finnie-Ansley et al., 2022). More complex programs, however, require learners to formulate clearer specifications and manage multiple program components, demands that are not eliminated by GAI assistance (Elgendy et al., 2026; Meske et al., 2025). The observed self-efficacy gradient therefore suggests that vibe coding may reduce syntactic barriers while leaving many higher-level programming demands intact (Elgendy et al., 2026; O'Connor et al., 2026).

The change in intentions to use GAI in education is reported as a secondary and exploratory outcome rather than as a principal contribution of the study, and it warrants careful interpretation. The pretest mean of 4.14 on a 5-point scale indicates that participants already reported strong intentions before the intervention, leaving limited room for measurable growth. Within technology acceptance frameworks, behavioral intention is associated with factors such as perceived usefulness, ease of use, and self-efficacy-related beliefs (Davis, 1989; Scherer et al., 2019). Previous studies have similarly reported favorable attitudes toward GAI among students and preservice teachers (Celik, 2023; Chan and Hu, 2023). Against this backdrop, the practical significance of a change of this size remains unclear. Behavioral intentions may also be less responsive to brief interventions than domain-specific self-efficacy. Although the increase was statistically significant, its practical importance appears limited. Scores rose by only 0.20 points on a five-point scale, from 4.14 to 4.34, suggesting that intentions were already high and remained so. Interpretation is further limited because full measurement invariance was not established, which means that the two administrations cannot be assumed to operate on exactly the same metric. This finding is therefore best treated as a secondary observation that contextualizes the self-efficacy results rather than as evidence that a brief vibe coding experience changes technology acceptance.

Beyond these specific outcomes, the findings contribute to ongoing discussions regarding the role of natural-language programming in computing education. Previous research has documented both the capabilities of code-generating models and the challenges associated with their educational integration (Finnie-Ansley et al., 2022; Prather et al., 2023). The present findings suggest that vibe coding may provide an accessible entry point to programming for learners who would otherwise be unlikely to engage with programming activities. This may be particularly relevant for preservice teachers, whose confidence with technology has been linked to later classroom use (Celik, 2023; Ertmer and Ottenbreit-Leftwich, 2010). At the same time, the present study assessed confidence rather than competence. Increased self-efficacy does not necessarily indicate improved programming skill or stronger ability to evaluate GAI-generated code (Prather et al., 2023). Kang and Huang (2026), for example, found that higher post-intervention coding self-efficacy was not significantly related to performance on a coding assessment. Pellas (2026) reported the opposite pattern, with preservice teachers showing lower coding confidence after GAI-mediated game making, which was interpreted as a recalibration following greater exposure to the demands of programming. These contrasting findings suggest that changes in confidence may depend on how much the learning tasks reveal the limitations of GAI support. The relatively contained tasks used in the present study may have provided fewer opportunities for such recalibration. This distinction may be especially important in vibe coding, where learners can generate functioning programs without fully engaging with the underlying code (Elgendy et al., 2026). Future research should therefore combine self-efficacy measures with performance-based assessments of programming outcomes.

The practical implications for teacher education should likewise be interpreted cautiously. Vibe coding appears to be a feasible instructional approach that can be incorporated into existing instructional technology courses, particularly when the goal is to reduce barriers to initial engagement with programming. However, outcomes extending beyond confidence, such as programming competence or critical evaluation of GAI-generated outputs, may require longer interventions and more comprehensive assessment strategies.

## Limitations and conclusion

Several limitations should be considered when interpreting these findings. First, the study employed a single-group pretest-posttest design without a control group. As a result, the observed changes cannot be attributed to the intervention, and alternative explanations such as maturation, repeated testing, regression to the mean, and other threats to internal validity cannot be ruled out. This concern is particularly relevant for programming self-efficacy because pretest scores were very low (e.g., a simple-task mean of 1.48 on a 7-point scale), leaving substantial room for improvement. Because pretest scores were close to the lower end of the scale, regression to the mean is likely to have contributed to the observed change. Other influences associated with the single-group design, including repeated testing and concurrent course experiences, also cannot be separated from the intervention. The reported effect sizes should therefore be interpreted as descriptions of within-sample change over the study period rather than as estimates of the effect of vibe coding. Future research should employ randomized or quasi-experimental designs with active or comparison groups to provide a more rigorous evaluation of effectiveness.

Second, both outcomes were assessed through self-report measures. Programming self-efficacy reflects participants’ confidence in their programming capabilities rather than their actual programming performance, and the two do not necessarily correspond. This distinction is particularly important in the present context because the intervention relied on GAI-supported code generation through natural-language prompting, whereas the CPSES items refer broadly to writing, debugging, and understanding programs. Consequently, posttest responses may partly reflect increased confidence in GAI-assisted programming workflows rather than equivalent gains in unaided programming competence. Participants may also have interpreted some items as referring to GAI-assisted program creation rather than independent programming ability. In addition, participants were aware that they were taking part in an intervention study, which may have increased the likelihood of demand characteristics or socially desirable responding. Future studies would benefit from complementing self-report measures with objective indicators of programming performance and from distinguishing unaided programming self-efficacy from GAI-assisted programming self-efficacy.

Third, the sample consisted of preservice teachers from four programs at a single university and was predominantly women (92.5%). This gender composition likely reflects the demographic characteristics of the participating programs, which are traditionally female-dominated in the Turkish higher education context. Consequently, the findings may not generalize to preservice teachers in computing-related or more gender-balanced fields, whose prior programming experience and baseline confidence may differ substantially. In addition, participants were enrolled in an elective course on artificial intelligence applications and may therefore have held more favorable attitudes toward GAI than the broader preservice teacher population, which may partly explain the high baseline intention scores. These characteristics limit the generalizability of the findings to other disciplines, institutions, and student populations.

Fourth, full measurement equivalence across the two occasions was not established for either instrument. The CPSES did not achieve acceptable configural fit, preventing further invariance testing, while the BIS supported metric but not scalar invariance. The pretest-posttest differences should therefore be interpreted as changes in observed scores rather than as evidence that equivalently measured constructs increased. It also remains possible that participants interpreted some items differently after the GAI-mediated programming experience.

Fifth, 28 of the 187 enrolled students (15.0%) did not provide paired data because they were absent from one of the two administrations. Because no records were retained for students who did not provide both administrations, the excluded and analyzed students could not be compared at the individual level. Systematic missingness therefore cannot be ruled out, and the analyzed sample may have over-represented students who were more engaged with the course.

Sixth, the intervention was relatively brief, with approximately six instructional hours delivered across three sessions, and only immediate posttest outcomes were assessed. It therefore remains unclear whether the observed changes would persist over time or translate into continued use of GAI-supported tools in educational practice. Seventh, the small increase in intentions to use GAI in education (dz = 0.29) should be interpreted in light of the high pretest level (M = 4.14 on a five-point scale), which left limited room for further change. Finally, participants’ interactions with the GAI system were not logged, so the extent of prompting and revision during the tasks could not be examined. Future studies should capture these process data to better understand how students engage with GAI-assisted programming.

In conclusion, programming self-efficacy scores among preservice teachers who participated in the four-week vibe coding intervention were substantially higher at posttest than at pretest. The difference was observed for both simple and complex tasks, although it was larger for simple tasks and confidence in complex programming remained lower. Because pretest scores were close to the lower end of the response scale, these differences should be interpreted as within-sample change rather than as estimates of the effect of vibe coding. Intentions to use GAI in education also increased, albeit to a lesser extent from an already high baseline. Taken together, the findings suggest that vibe coding may offer an accessible entry point to programming by reducing barriers associated with traditional syntax-focused instruction. At the same time, the results indicate that confidence gained through short-term exposure should not be equated with programming competence and that further research using controlled designs and performance-based measures is needed. Nor should it be assumed to reflect unaided programming ability, given that the intervention centered on GAI-supported code generation. More broadly, the study highlights the potential of GAI-supported programming approaches as a tool for introducing computational experiences to future educators.

## Data Availability

The raw data supporting the conclusions of this article will be made available by the authors, without undue reservation.
