# Supplementary material for: Vibe coding with preservice teachers: a single-group pretest-posttest pilot study of programming self-efficacy and intentions to use generative AI in education
Source: Front Psychol. 2026 Sep 28;17:1957111. doi: 10.3389/fpsyg.2026.1957111 (PMC13655020; doi:10.3389/fpsyg.2026.1957111)
Supplement: Supplementary file 1 [file Supplementary_file_1.DOCX]

**Supplementary Material**

*Vibe coding with preservice teachers: A single-group pretest–posttest pilot study of programming self-efficacy and intentions to use generative AI in education*

Frontiers in Psychology

This file contains the instructional materials required to reproduce the intervention (S1–S3) and the full output of the analyses added during revision (S4–S6). Section numbers correspond to the references given in the main manuscript.

**S1. Instructor prompt examples and prompting strategy**

The prompts below are those demonstrated by the instructor during the three instructional sessions. Participants were not required to reproduce them verbatim; they were presented as models of how to state a problem to a generative AI system in natural language.

**S1.1 Session 1 — orientation and first prompts**

**Prompt 1 — Simple calculation**

Turkish: “İki sayıyı kullanıcıdan alan, bu sayıların toplamını hesaplayan ve sonucu ekranda gösteren basit bir program oluştur.”

English: “Create a simple program that takes two numbers from the user, calculates their sum, and displays the result on the screen.”

**Prompt 2 — Formatted output**

Turkish: “Kullanıcıdan adını ve yaşını alan ve ekranda ‘Merhaba [isim], [yaş] yaşındasın.’ şeklinde bir mesaj gösteren bir program oluştur.”

English: “Create a program that asks the user for their name and age and displays the message ‘Hello [name], you are [age] years old.’”

**Prompt 3 — Extending the initial program**

Turkish: “Bu programı geliştir. Kullanıcının doğum yılını da hesaplasın ve ekranda adını, yaşını ve tahmini doğum yılını birlikte göstersin.”

English: “Extend this program so that it also calculates the user’s year of birth and displays their name, age, and estimated year of birth together.”

**Prompt 4 — First prompt-refinement example**

Turkish: “Program istediğim gibi çalışmadı. Sorunu belirle ve programın doğru çalışması için gerekli düzeltmeleri yap.”

English: “The program did not work as intended. Identify the problem and make the necessary corrections so that the program works correctly.”

**S1.2 Session 2 — prompting for simple programs**

**Prompt 1**

Turkish: “Kullanıcıdan üç sayı alan, bu sayıların ortalamasını hesaplayan ve sonucu ekranda gösteren bir program oluştur.”

English: “Create a program that takes three numbers from the user, calculates their average, and displays the result on the screen.”

**Prompt 2**

Turkish: “Kullanıcıdan bir sayı alan ve bu sayının tek mi çift mi olduğunu belirleyerek sonucu ekranda gösteren bir program oluştur.”

English: “Create a program that takes a number from the user, determines whether it is odd or even, and displays the result.”

**Prompt 3**

Turkish: “Kullanıcıdan bir sınav notu alan ve nota göre öğrencinin başarılı veya başarısız olduğunu ekranda gösteren basit bir program oluştur. 50 ve üzerindeki notları başarılı kabul et.”

English: “Create a simple program that takes an exam score from the user and displays whether the student passed or failed. Consider scores of 50 or above as passing.”

**Prompt 4**

Turkish: “Bu programı geliştirerek kullanıcı yanlış veya geçersiz bir değer girdiğinde hata vermek yerine kullanıcıya uygun bir uyarı mesajı göstermesini sağla.”

English: “Improve this program so that, when the user enters an incorrect or invalid value, it displays an appropriate warning message instead of producing an error.”

**S1.3 Session 3 — staged prompting for complex programs**

In the third session participants were introduced to a staged prompting strategy in which the model was first asked to analyse the problem and state the intended functionality, and only then asked to produce code. The instructor demonstrated the strategy using the following prompts.

**(a) Analysis prompt**

Turkish: “Bir öğrencinin girdiği ders notlarını kaydeden, bu notların ortalamasını hesaplayan ve ortalamaya göre öğrencinin başarı durumunu gösteren bir program geliştirmek istiyorum. Henüz kod yazma. Önce problemi analiz et, programın sahip olması gereken temel işlevleri belirle ve programın nasıl çalışacağını adım adım açıkla.”

English: “I want to develop a program that records the course grades entered for a student, calculates the average of these grades, and displays the student’s achievement status based on the average. Do not write any code yet. First analyse the problem, identify the main functions the program should have, and explain step by step how the program should work.”

**(b) Code-generation prompt**

Turkish: “Şimdi belirlediğin işlevlere ve çalışma adımlarına göre programın kodunu oluştur. Program kullanıcıdan gerekli bilgileri alsın, hesaplamaları yapsın ve sonuçları anlaşılır bir şekilde ekranda göstersin.”

English: “Now generate the program code according to the functions and steps you identified. The program should obtain the necessary information from the user, perform the calculations, and display the results clearly.”

**(c) Follow-up prompt when the output did not run**

Turkish: “Program çalıştırıldığında hata veriyor veya beklenen çıktıyı üretmiyor. Mevcut programı incele, sorunun nedenini belirle ve programın amaçlandığı şekilde çalışması için gerekli düzeltmeleri yap.”

English: “When the program is run, it produces an error or does not generate the expected output. Examine the current program, identify the cause of the problem, and make the necessary corrections so that the program works as intended.”

**S1.4 Model and settings**

Programming activities were completed in Python using Google AI Studio, accessed through a standard web browser. The model used was Gemini 2.5 Flash. All model settings were retained at their default values; no parameters were modified, and no custom system instruction was configured, so that the environment would match what participants could reproduce independently outside class.

**S2. Programming assignments and completion criteria**

Eight assignments were set in total: three simple tasks following the second session and five complex tasks following the third session. Completion was recorded on a binary basis. An assignment was counted as complete when the submitted screenshot showed the program running and producing the specified output; no rubric assessing code quality was applied.

*Table S2. Full text of the programming assignments*

| **#** | **Type** | **Assignment text** | **Completion criterion** |
| --- | --- | --- | --- |
| 1 | Simple | Create a program that displays your first and last name on the screen. | The submitted screenshot shows the program running and correctly displaying the student’s first and last name. |
| 2 | Simple | Create a program that calculates the arithmetic mean of three numbers. | The submitted screenshot shows three numerical values being entered and the program displaying their calculated arithmetic mean. |
| 3 | Simple | Create a program that calculates the arithmetic mean of any given series of numbers. | The submitted screenshot shows a series of numerical values being entered and the program displaying the calculated arithmetic mean. |
| 4 | Complex | Develop a personal income-and-expense recording and analysis application. The application should allow users to enter income and expenses, organize transactions, and provide an overview of their financial records. | The submitted screenshot shows the application running, recording income and expense data, and displaying a summary of the recorded transactions. |
| 5 | Complex | Extend the income-and-expense application so that users can classify transactions into categories and review the distribution of their spending across categories. | The submitted screenshot shows categorized financial records and a functioning summary or visualization of the category distribution. |
| 6 | Complex | Develop an analysis component that uses the recorded financial data to provide the user with summaries of income, expenditure, and spending patterns. | The submitted screenshot shows the application processing the entered data and displaying financial analyses or summary statistics. |
| 7 | Complex | Improve the application by identifying and correcting functional problems and adding features that make the program easier and more useful to operate. | The submitted screenshot shows the revised application running successfully with the required functions and improvements implemented. |
| 8 | Complex | Produce a final, integrated version of the application with a structured dashboard that combines data entry, stored records, analyses, and summary information in a usable interface. | The submitted screenshot shows the completed application running with an integrated dashboard and the required components functioning together. |

Note. Assignments were submitted through the institutional learning management system as screenshots of the executed program. Interaction logs were not collected, so the number of prompts used, the extent of participant modification of generated code, and participants' verification procedures were not recorded.

**S3. Representative student submissions**

The examples below illustrate the range of submissions received. Identifying information has been removed. The screenshots are presented only to illustrate the types of programming output participants submitted; they were not used to assess code quality.

**Example 1. Simple programming task**

Assignment attempted: Assignment 3 — create a program that calculates the arithmetic mean of any given series of numbers.

Prompt used: “Verilen herhangi bir sayı dizisinin ortalamasını hesaplayan bir program yaz.” (“Create a program that calculates the arithmetic mean of any given series of numbers.”)

Resulting output: the submitted program accepted a series of numerical values and displayed the calculated arithmetic mean together with summary information for the entered values.

*Figure S1. Representative output for a simple programming assignment.*

**Example 2. Complex personal finance application**

Assignment attempted: Assignment 4 — develop a personal income-and-expense recording and analysis application that records transactions and provides a summary of the user’s financial data.

Prompting approach: the submission documented the staged sequence introduced in the third session — problem analysis, generation of an initial working version, explanation of the code, debugging, improvement, and final refinement.

Resulting output: the program displayed income and expense totals together with visual summaries of spending, including category distribution and expenditure analysis.

*Figure S2. Representative output from a staged complex programming submission.*

**Example 3. Complex dashboard-style application**

Assignment attempted: Assignment 8 — produce a final, integrated version of the application combining data entry, stored records, analyses, and summary information in a usable interface.

Prompting approach: the submission followed the same staged workflow used for the other complex activities, progressing from problem analysis and an initial working version to debugging, improvement, and a more polished final version.

Resulting output: the final submission presented a dashboard-style interface integrating summary indicators and analytical information in a single view.

*Figure S3. Representative dashboard-style output from a complex programming submission.*

Note. The examples were selected to show the range from a basic calculation task to multi-component applications. Only information visible in the submitted materials was used to describe them.

**S4. Confirmatory factor analysis: model fit and standardized loadings**

Confirmatory factor analyses were estimated separately at pretest and posttest for the Computer Programming Self-Efficacy Scale (CPSES) and the Behavioural Intention Scale (BIS). Chi-square statistics and degrees of freedom are reported so that the elevated RMSEA values obtained for three of the four models can be evaluated against item-level fit rather than interpreted in isolation.

*Table S4.1. Confirmatory factor analysis model fit*

| **Model** | **χ²** | **df** | **χ²/df** | **CFI** | **GFI** | **RMSEA** | **SRMR** |
| --- | --- | --- | --- | --- | --- | --- | --- |
| CPSES pretest | 97.97 | 23 | 4.26 | .92 | .89 | .14 | .06 |
| CPSES posttest | 50.94 | 23 | 2.22 | .98 | .94 | .09 | .06 |
| BIS pretest | 5.96 | 4 | 1.49 | .99 | .99 | .06 | .02 |
| BIS posttest | 14.25 | 4 | 3.56 | .97 | .97 | .13 | .04 |

Note. N = 159 at each occasion. Fit indices already reported in Table 2 of the main manuscript are reproduced here alongside the chi-square statistics and degrees of freedom. Of the four models, only the BIS pretest model met every criterion adopted in advance; the CPSES pretest model exceeded the RMSEA threshold and fell below the GFI criterion, and the CPSES posttest and BIS posttest models exceeded the RMSEA threshold. CFI = comparative fit index; GFI = goodness-of-fit index; RMSEA = root mean square error of approximation; SRMR = standardized root mean square residual.

*Table S4.2. Standardized factor loadings at pretest and posttest*

| **Scale / factor** | **Item** | **Pretest λ** | **Posttest λ** |
| --- | --- | --- | --- |
| CPSES — Simple tasks | 1 | .84 | .83 |
|  | 2 | .92 | .99 |
|  | 3 | .91 | .95 |
| CPSES — Complex tasks | 4 | .69 | .74 |
|  | 5 | .66 | .86 |
|  | 6 | .74 | .91 |
|  | 7 | .72 | .85 |
|  | 8 | .81 | .80 |
|  | 9 | .71 | .61 |
| BIS | 1 | .82 | .62 |
|  | 2 | .82 | .74 |
|  | 3 | .82 | .87 |
|  | 4 | .81 | .82 |
|  | 5 | .68 | .61 |

Note. All loadings are completely standardized and significant at p < .001. Every loading exceeded .60 at both occasions. The lowest values occurred at posttest for CPSES item 9 (.61), which refers to maintaining focus despite distractions rather than to a programming operation, and for BIS items 1 (.62) and 5 (.61). Item-level results therefore did not indicate clearly problematic indicators. The elevated RMSEA values in Table S4.1 should be interpreted alongside the standardized loadings and the relatively low degrees of freedom rather than in isolation.

**S5. Longitudinal measurement invariance**

**S5.1 Procedure**

Because the same instruments were administered on two occasions, longitudinal measurement invariance was examined before pretest–posttest mean differences were interpreted. The pretest and posttest indicators were specified within a single model, with the residuals of the same item allowed to correlate across occasions, so that the dependency between the two observations from each participant was modelled rather than ignored (Little, 2013). Configural, metric (equal loadings) and scalar (equal intercepts) models were then compared sequentially. All models were estimated on the 159 participants with complete paired data.

Because the chi-square difference test is sensitive to sample size, invariance was evaluated using changes in approximate fit indices. A decrease in CFI no greater than .010, together with an increase in RMSEA no greater than .015, was treated as supportive of invariance (Cheung & Rensvold, 2002). Both criteria had to be met; where the indices diverged, the decision followed this rule rather than the most favourable index.

**S5.2 Model fit**

*Table S5. Longitudinal measurement invariance models*

| **Scale / model** | **χ²** | **df** | **CFI** | **TLI** | **RMSEA** | **ΔCFI** | **ΔRMSEA** | **Decision** |
| --- | --- | --- | --- | --- | --- | --- | --- | --- |
| CPSES |  |  |  |  |  |  |  |  |
| Configural | 351.01 | 120 | .901 | .873 | .110 | — | — | Not supported |
| Metric | 416.84 | 127 | .875 | .850 | .120 | −.026 | +.010 | Not interpreted |
| Scalar | — | — | — | — | — | — | — | Not estimated |
| BIS |  |  |  |  |  |  |  |  |
| Configural | 78.32 | 29 | .942 | .910 | .104 | — | — | Partially acceptable |
| Metric | 80.36 | 33 | .944 | .924 | .095 | +.002 | −.009 | Supported |
| Scalar | 97.81 | 38 | .929 | .916 | .100 | −.015 | +.005 | Not supported |

Note. N = 159. Pretest and posttest indicators were specified within a single model with correlated residuals for the same item across occasions. Δ values compare each model with the preceding, less constrained model in the same block. A model was recorded as supported when CFI decreased by no more than .010 and RMSEA increased by no more than .015; both criteria were required. The CPSES model specifies the simple-task and complex-task factors at both occasions across 18 indicators. The BIS configural model is recorded as partially acceptable because it met the CFI and TLI criteria but not the RMSEA criterion; the metric and scalar comparisons are evaluated against it on that basis, as explained in S5.3. Because acceptable configural fit was not obtained for the CPSES, the metric model estimated for that instrument is reported for completeness but was not interpreted as a test of invariance, and the scalar model was not estimated.

**S5.3 Results**

For the CPSES, the configural model did not achieve acceptable fit (χ²(120) = 351.01, CFI = .901, TLI = .873, RMSEA = .110). The CFI only marginally reached the adopted criterion, the TLI fell below it, and the RMSEA was well above the threshold. Because the configural model imposes no equality constraints across occasions, this indicates that the measurement model as specified is not adequately supported at both administrations. The metric model was estimated and is reported in Table S5 for completeness, but it was not interpreted as a test of invariance, and the scalar model was not estimated: with the configural baseline unsupported, comparisons against it would not be informative about equivalence.

Two features of the data are likely to have contributed. Pretest item means ranged from 1.23 to 2.13 on a seven-point scale, with the large majority of participants selecting the lowest response option, so that little covariance among items remained to be modelled. The model also estimates a substantial number of parameters relative to the available sample.

For the BIS, whose pretest responses were not subject to a floor effect, the configural model met the CFI and TLI criteria (χ²(29) = 78.32, CFI = .942, TLI = .910), although the RMSEA (.104) exceeded the .08 threshold. On that baseline, metric invariance was supported: constraining the loadings to equality across occasions did not degrade fit on either criterion (ΔCFI = +.002, ΔRMSEA = −.009), and both indices in fact improved. Adding the intercept constraints degraded fit beyond the adopted criteria (ΔCFI = −.015, ΔRMSEA = +.005), so scalar invariance was not supported; the shortfall is on the CFI criterion alone and is modest in magnitude.

One qualification applies to the BIS sequence and is stated here rather than left to the reader. Because the configural baseline did not meet the RMSEA criterion, the invariance comparisons for that instrument rest on a baseline model whose fit is adequate but not uniformly strong. Changes in fit indices are informative about the effect of the added constraints, but they do not compensate for the fit of the model against which those constraints are evaluated.

**S5.4 Interpretive consequences**

Full measurement equivalence across the two occasions was therefore not established for either instrument, although for different reasons: for the CPSES because acceptable configural fit was not obtained, so that no test of invariance could be conducted at all, and for the BIS because scalar invariance was not supported once metric invariance had been established. The pretest–posttest differences reported in the main manuscript are accordingly presented as changes in observed scores rather than as changes in constructs measured equivalently on the two occasions, and they support the claim that participants reported higher confidence and stronger intentions at posttest rather than the stronger claim that equivalently measured constructs increased.

The possibility that participants interpreted the items differently after four weeks of GAI-mediated programming cannot be ruled out, and this is stated in the Discussion and Limitations of the main manuscript. It is not the only explanation available: for the CPSES, the floor-level pretest distributions and the number of parameters estimated relative to the sample are sufficient in themselves to account for the result, and the present data do not distinguish among these accounts.

**S5.5 Partial invariance**

Partial invariance models were not pursued. Freeing loadings or intercepts on the basis of modification indices within a single sample is an exploratory specification search that capitalises on chance features of that sample, and the resulting model requires cross-validation in an independent sample before it can be relied upon (MacCallum, Roznowski, & Necowitz, 1992). Reporting a partially invariant model estimated and evaluated in the same data would overstate the security of the measurement basis for the comparisons that follow.

Partial scalar invariance was not examined for the BIS. The scalar model showed a modest deterioration in fit that nevertheless exceeded the adopted CFI criterion (ΔCFI = −.015 against a criterion of −.010), while ΔRMSEA remained within its criterion. However, identifying and freeing non-invariant intercepts on the basis of modification indices would have constituted a post hoc, sample-specific model modification. Because such a model could not be cross-validated in independent data, partial invariance was not pursued. Accordingly, the pretest–posttest difference for the BIS continues to be interpreted as a change in observed scores rather than as a change in an equivalently measured latent construct.

**S6. Multiplicity-corrected p-values**

Three pretest–posttest comparisons were conducted, corresponding to the two hypotheses specified in advance of data collection. Because the outcomes were pre-specified rather than selected after inspection of the data, a correction was not strictly required; corrected values are reported so that readers can verify that the substantive conclusions do not depend on how multiplicity is handled.

*Table S6. Uncorrected and corrected p-values for the three pretest–posttest comparisons*

| **Outcome** | **t (158)** | **Uncorrected p** | **Bonferroni threshold** | **BH-adjusted p** | **Significant** |
| --- | --- | --- | --- | --- | --- |
| Self-efficacy, simple tasks | 33.05 | < .001 | .017 | < .001 | Yes |
| Self-efficacy, complex tasks | 21.64 | < .001 | .017 | < .001 | Yes |
| Intentions to use GAI | 3.68 | < .001 | .017 | < .001 | Yes |

Note. m = 3 comparisons. Bonferroni: the threshold is α/m = .05/3 = .017; equivalently, p × m may be compared with .05, which gives the same decision. Benjamini–Hochberg: p-values are ranked in ascending order (i = 1, 2, 3), the critical value for rank i is (i/m)q, giving .017, .033 and .050 at q = .05, and the largest i satisfying pᵢ ≤ (i/m)q determines significance for that rank and all lower ranks; adjusted values are pᵢ × m/i, made monotone from the highest rank downward. The two self-efficacy comparisons yielded p-values below the precision reported by the analysis software and are shown as < .001; the comparison for intentions to use GAI, which is the closest to any threshold, had an uncorrected value of p = .0003 and a Bonferroni-corrected value of p = .0010, both well below the criterion. Test statistics correspond to those reported in Table 4 of the main manuscript. Wilcoxon signed-rank tests produced the same pattern of results.

**Supplementary references**

Benjamini, Y., & Hochberg, Y. (1995). Controlling the false discovery rate: A practical and powerful approach to multiple testing. Journal of the Royal Statistical Society: Series B (Methodological), 57(1), 289–300.

Cheung, G. W., & Rensvold, R. B. (2002). Evaluating goodness-of-fit indexes for testing measurement invariance. Structural Equation Modeling: A Multidisciplinary Journal, 9(2), 233–255.

Little, T. D. (2013). Longitudinal structural equation modeling. Guilford Press.

MacCallum, R. C., Roznowski, M., & Necowitz, L. B. (1992). Model modifications in covariance structure analysis: The problem of capitalization on chance. Psychological Bulletin, 111(3), 490–504.
